# Serum-Based Biomarkers in Neurodegeneration and Multiple Sclerosis

**DOI:** 10.3390/biomedicines10051077

**Published:** 2022-05-06

**Authors:** Patrizia LoPresti

**Affiliations:** Department of Psychology, The University of Illinois at Chicago, 1007 West Harrison Street, Chicago, IL 60607, USA; patrizia.lopresti.22@gmail.com

**Keywords:** neurodegeneration, cognition, progressive multiple sclerosis, biomarkers, diagnosis, treatment, prognosis, personalized medicine, cytoskeleton, synapse

## Abstract

Multiple Sclerosis (MS) is a debilitating disease with typical onset between 20 and 40 years of age, so the disability associated with this disease, unfortunately, occurs in the prime of life. At a very early stage of MS, the relapsing-remitting mobility impairment occurs in parallel with a progressive decline in cognition, which is subclinical. This stage of the disease is considered the beginning of progressive MS. Understanding where a patient is along such a subclinical phase could be critical for therapeutic efficacy and enrollment in clinical trials to test drugs targeted at neurodegeneration. Since the disease course is uneven among patients, biomarkers are needed to provide insights into pathogenesis, diagnosis, and prognosis of events that affect neurons during this subclinical phase that shapes neurodegeneration and disability. Thus, subclinical cognitive decline must be better understood. One approach to this problem is to follow known biomarkers of neurodegeneration over time. These biomarkers include Neurofilament, Tau and phosphotau protein, amyloid-peptide-β, Brl2 and Brl2-23, N-Acetylaspartate, and 14-3-3 family proteins. A composite set of these serum-based biomarkers of neurodegeneration might provide a distinct signature in early vs. late subclinical cognitive decline, thus offering additional diagnostic criteria for progressive neurodegeneration and response to treatment. Studies on serum-based biomarkers are described together with selective studies on CSF-based biomarkers and MRI-based biomarkers.

## 1. Introduction

Multiple Sclerosis (MS) affects approximately 2.2 million people worldwide, with prevalence in white northern European descendants, and affecting more women than men [1,2,3]. The onset of this debilitating disease is usually between 20 and 40 years, so the disability associated with MS, unfortunately, occurs in the prime of life, making MS the most common neurological disability in young adults. While the cause of MS has not been determined, combinations of epidemiology, genetics, and environment are thought to contribute. 

The risk of developing MS increases with low exposure to the sun, viral infections, smoking, genetic susceptibility, and location north of the equator. Recent work has shown that the high prevalence of Epstein-Barr virus is associated with MS [4,5]. Overall MS disease results from multiple agents priming the immune system over time until the immune system starts to attack and damage the central nervous system (CNS) [3,5]. Each patient has unique inflammatory responses as well as interactions between the innate and acquired immune systems. The involvement of inflammation and complex interactions between innate and acquired immunity makes the disease unique in each patient with different impacts on the CNS.

The symptoms of MS include fatigue, depression, vertigo, visual problems, cognitive dysfunction, muscle-related symptoms, bowel and bladder symptoms, and sexual dysfunctions. The diagnosis of MS is made using a combination of tests, such as magnetic resonance imaging, spinal tap, and evoked potential analysis. In addition, the expanded disability status scale is widely used to measure the degree of disability [6,7]. The use of diagnostic tools to detect subclinical neuronal functional decline is imperative; a new MS classification should address the degree of neurodegeneration, which would facilitate correct enrollment into clinical trials.

MS is a complex disease, having independent and interconnected components [8]. The subclinical mobility defect detected with the highly sensitive glove test [9] was not included in the definition of relapsing-remitting (RR)-MS, whereas Benedict et al. [10] described some recovery of cognitive function throughout the disease; however, that recovery was partial, which inevitably would result in a progressive decline of cognitive functions. 

The main therapeutic challenge is that within an apparent clinically homogenous group of MS patients, the underlying neurodegeneration might be at earlier or later stages. Indeed, detecting such subclinical (silent) progression would aid in MS diagnosis, treatment with new therapeutics, and prognosis [11,12]. Neurons damaged during MS release molecules into the extracellular CNS compartments where they can be re-internalized and/or destroyed [13]. The extent of re-internalization and/or destruction would determine the levels of biomarkers detected in cerebrospinal fluid (CSF) and blood samples. CSF sampling offers a more accurate analysis [13,14]; however, CSF sampling is not easy for patients. In contrast, blood samples can be obtained from patients with little stress or discomfort, and serum-based biomarkers are a less expensive means to assess neuronal degeneration. These tests can be easily repeated at a low cost. In contrast, Magnetic Resonance Imaging (MRI)-based biomarkers can only be performed at select clinical medical centers, in addition to being expensive and time-consuming.

A personalized medicine approach is greatly needed as a set of biomarkers could flag each patient affected by MS before and after treatment [15,16]. Biomarkers provide a platform for identifying disease mechanisms and pharmacological targets. Exploratory analysis and validation are some of the steps required to validate biomarkers for routine clinical practice [13,14]. The combined use of several biomarkers could better elucidate the underlying mechanisms during neurodegeneration. Biomarkers of axonal and neuronal degeneration could include NFL, tau, P-tau, β-amyloid 1-42, Bri2 (Integral membrane protein 2B), Bri2-23 (generated from Bri2), the neuronal mitochondrial metabolite NAA (N-acetyl aspartate), and 14-3-3. At the same time, a biomarker of T-cell-mediated autoimmunity such as Osteopontin could be added to further analyze disease activity [17,18,19,20,21,22,23,24,25,26,27,28,29]. In addition to the enzyme-linked immunosorbent assay (ELISA), new technologies with increased sensitivity and accuracy have been developed, including electrochemiluminescence (ECL), single-molecule array (SIMOA) technology, immunomagnetic reduction, immuno-precipitation/mass spectrometry, and dried plasma spot [30].

It is important to determine the ratio of CSF/plasma of selective biomarkers in disease models and upon treatment with various therapeutics. The widely used experimental autoimmune encephalomyelitis (EAE) mice should be used in parallel with additional models, including the model of MS where oligodendrocyte death results in immune-mediated CNS demyelination [31]. In addition, the Non-Obese Diabetic (NOD)-EAE model could also be useful since this model mimics RR-MS becoming Secondary Progressive (SP)-MS [32].

**Figure 1:**
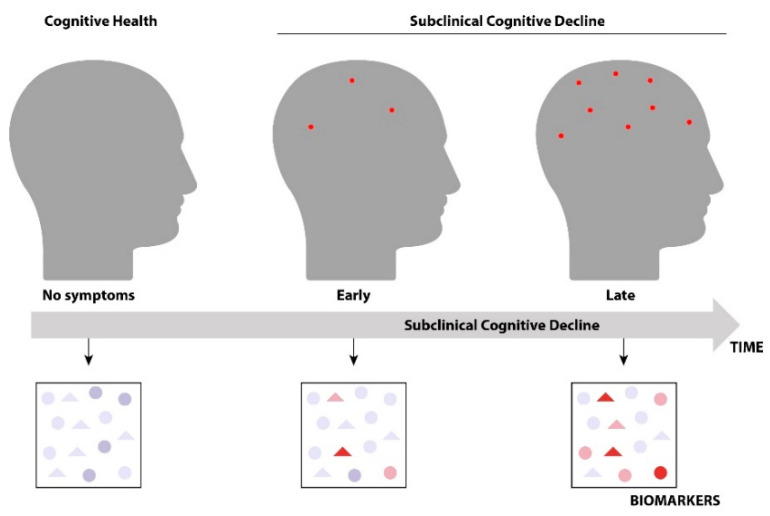
Biomarkers during Subclinical Cognitive Decline. During the subclinical cognitive decline preceding progressive multiple sclerosis, the patients are in early or late subclinical decline, i.e., far from, or closer to, developing progressive multiple sclerosis. A set of distinct biomarkers at these two-time points can provide insight into both diagnosis and treatment. The red dots represent synaptic dysfunction and neurodegeneration, whereas the panels with biomarkers indicate representative differences in biomarkers and distinctive signatures.

Synapses are believed to be an early target of MS disease, driving processes that lead to permanent neuronal loss [33,34,35,36,37,38,39,40,41,42]. Such an insidious foe for the synapses and neurons at the beginning of disease requires insight and targeted therapeutics. The United States Food and Drug Administration (FDA) has approved drugs that regulate immune-mediated inflammation. However, subclinical synaptic alterations are largely unmodified by current approaches, and a set of biomarkers could be used to characterize early vs. late neurodegeneration. Understanding where a patient is along the subclinical neurodegeneration pathway could be a critical tool for guiding drug enrollment in clinical studies (Figure 1).

**Figure 2:**
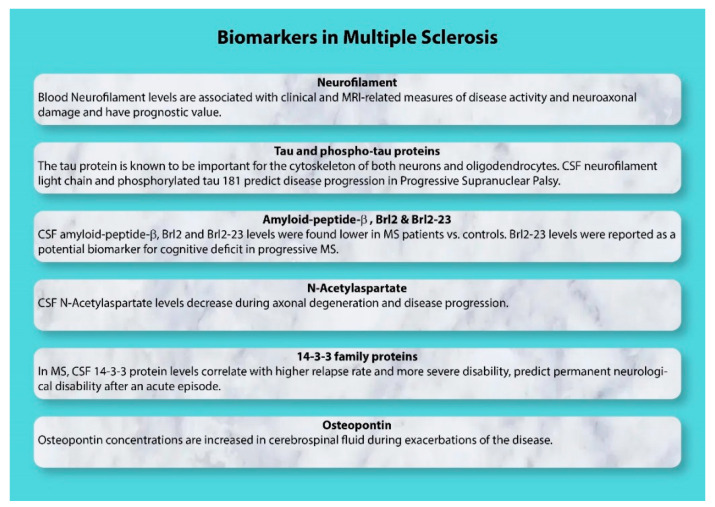
Biomarkers in Multiple Sclerosis. Biomarkers of neurodegeneration and inflammation are indicated. Neurodegeneration biomarkers include Neurofilament, Tau and phosphotau protein, amyloid-peptide-β, Brl2 and Brl2-23, N-Acetylaspartate, and 14-3-3 family proteins, whereas Osteopontin is an inflammation biomarker.

It is crucial to determine normal and pathological values of biomarkers (Figure 2) along with their specific fold-increase over longitudinal patient analysis [13,27,43]. Kappos et al. [44] have proposed using a roving reference value in prospective studies rather than a fixed value. Further, the distinction between biomarker expression during disease activity and progression must be understood in the context of underlying events, such as increased protein turnover vs. increased protein levels due, for example, to cytoskeletal disruption.

## 2. Neurofilaments

Neurofilaments (NFs) are proteins of the axonal cytoskeleton that are critical for intracellular transport and the health of the axons [45,46,47]. Neurofilaments consist of NF-light (L), -middle (M), and heavy (H) chains [48,49]. NF-L is a soluble and abundant component of neuronal axons. 

NF-L is the most abundant and soluble, whereas NF-H is the largest [13]. Both NF-H and NF-M regulate axonal diameter based on their phosphorylation levels. Highly phosphorylated NF-H is believed to indicate an axonal injury and may also increase during the progression of neurological diseases [50,51]. Healthy individuals have low levels of NF-L in the blood, whereas high levels of NF-L are detected in degenerative diseases, including Amyotrophic lateral sclerosis (ALS), MS, and Alzheimer’s disease (AD) [52,53,54]. 

NF-L levels correlate with axonal damage and with cognitive performance [55,56]. Studies on NF-L in AD could offer insight into approaches to neurodegeneration in MS because elevated NF-L levels in CSF and plasma indicate a neuronal injury and appear to be promising markers of AD severity and progression [57,58]. Of note, NF-L levels are increased in pre-symptomatic and early symptomatic stages of AD and correlate with cognitive decline, progression of brain atrophy, and decreased survival. Furthermore, the use of ultrasensitive biomarker assays such as SIMOA has allowed the quantitation of low NF-L levels in blood samples. Plasma or serum NF-L levels differentiate pre-symptomatic and early symptomatic AD from controls in studies of familial and sporadic AD, accurately predicting rates of disease progression over time [46,59]. 

NF-L increases during the acute phase of MS and high N-FL levels correlate with disease progression and/or conversion of RR-MS to SP-MS [60,61,62,63,64,65,66]. In contrast, the concentration of NF-L did not correlate with gender, age, or disease duration [13,67]. Blood NF-L levels are associated with clinical and MRI-related measures of disease activity and neuroaxonal damage and have prognostic value. Work by Kuhle et al. [68] supports the utility of blood NF-L as an easily accessible biomarker of disease evolution and treatment response. Of interest, the CSF NF-L chain predicts 10-year clinical and radiologic worsening in multiple sclerosis [69,70]. Lycke et al. [70] also showed that the CSF NF-L protein is a potential marker of activity in multiple sclerosis. Immunosuppressive therapy reduces axonal damage, and NF-L levels in CSF are a potential marker for treatment efficacy [68,71]. Further, diffusely abnormal white matter in clinically isolated syndrome, a first clinical episode compatible with MS, is associated with parenchymal loss and elevated neurofilament levels [72], and NF-L levels are associated with chronic white matter inflammation [73]. Recent important work has shown that antibodies to NF-L are potential MS biomarkers [74], and new MRI activity and NF-L levels effectively monitor MS patients [75]. In summary, NF-L is recognized as having an important value in both research and clinical trial settings [76]. Barro et al. [53] showed that CFLs predict disease worsening and brain and spinal cord atrophy in MS. Further, NF-L in CSF and serum is a sensitive marker for axonal white matter injury [77]. Bridel et al. [78] showed that NF-L is associated with progression in Natalizumab-treated patients with RR-MS, whereas Kuhle et al. [79] showed a reduction in sNF-L following early alemtuzumab treatment in RR-MS patients. Cantó et al. [80] showed an association between sNF levels and long-term disease course, and Kuhle et al. [81] demonstrated a correlation between high NF-L levels and long-term outcomes. Additional work is required to investigate whether selected drugs aimed at neuroprotection decrease NF-L levels in MS patients. Table 1 summarizes neurofilament detection in various types of human samples using a variety of assays, including ELISA, ECL, and SIMOA.

## 3. Total and Phosphorylated Tau

Tau protein is important for the cytoskeleton of both neurons and oligodendrocytes [114,115,116,117]. Neurons and oligodendrocytes depend on efficient intracellular transport to accomplish tasks such as synaptic transmission and myelination, which are both essential for CNS health. Abnormally phosphorylated (P-)tau, a hallmark of CNS degenerative diseases, was found in chronic EAE and progressive MS [118,119]. 

Abnormally phosphorylated P-tau in neurons and/or oligodendrocytes causes cells to deteriorate, which would enhance disease progression [115,116,117,120,121]. Histological studies have clearly shown increased levels of P-tau in progressive MS [117,118,119]. Surprisingly, studies on total tau and P-tau as MS biomarkers have yielded contradictory data, perhaps due to a high sensitivity to tau protein degradation during improper samplings [13]. Indeed, some studies have reported higher levels of tau/P-tau in MS than in controls; however, others could not confirm this finding. Similar contradictions were reported regarding the correlation between a disability, inflammation, and age or MS disease duration [13,122]. Prednisolone effectively reduces plasma tau and P-tau in EAE rats [123]. Of interest, Rojas et al. [124] showed that CSF NF-L and tau phosphorylated at threonine 181 (P-tau181) predict disease progression in Progressive Supranuclear Palsy. It is imperative to investigate whether drugs aimed at neuroprotection in MS decrease NF-L levels in association with decreased tau phosphorylated at a specific site. Because cerebrospinal Tau levels predict early disability in MS [21], NF-L and tau have been proposed as biomarkers to monitor prognosis and treatment response, distinguishing different MS subtypes [125]. 

## 4. Amyloid-Peptide-β, Bri2, and Bri2-23

Amyloid precursor protein (APP) functions as cargo transported by fast anterograde axonal transport, whereas APP builds up in the cell body during diseases of axons [43]. APP, an integral membrane protein, is cleaved by the integral membrane aspartyl protease BACE1 (β-site APP-cleaving enzyme 1) into three fragments (α-sAPP, β-sAPP, and Aβ42) [13]. CSF levels of these fragments were lower in MS patients than in controls. Interestingly, the Bri2 and Bri2-23 molecules interact with APP and regulate amyloid-peptide-β (Aβ)42 cleavage and aggregation in vivo. Bri2 is a transmembrane protein and Bri2-23 is a peptide cleaved from Bri2. Because Bri2-23 levels have been suggested as a potential biomarker for cognitive deficit in progressive MS, studies should assess the effect of drugs aimed at neuroprotection on APP-derived proteins and on Bri2 and Bri2-23. Finally, Aβ42 does not correlate with age or disease duration [13,126,127]. 

The extent of APP expression appears to correlate with histopathological lesions, suggesting that APP detection is a sensitive marker for MS disease progression [126,128]. Insight from studies in AD could help to better tackle the challenges of MS biomarkers. High levels of CSF tau and/or P-tau181, and low levels of CSF Aβ42, are detected in the pre-symptomatic stages of AD disease [129]. Importantly, the ratio of CSF tau/Aβ42 or CSF P-tau181/Aβ42 indicates the progression of AD pathology, which could also predict cognitive impairment in cognitively normal individuals [130,131,132]. The utility of the ratio of CSF levels Aβ42/Aβ40 or Aβ42/Aβ38 provides a better diagnostic value than the total levels of CSF Aβ42 [131]. Tau phosphorylation at selective sites, in particular, levels of CSF P-tau231 (threonine 231) and P-tau181, but not CSF P-tau199 (serine tau 199), differentiate AD from non-AD dementias [131]. CSF P-tau231 aids in differentiating AD from frontotemporal dementia and CSF P-tau181 differentiates AD from Lewy body dementia (LBD) [131]. In addition, the levels of CSF P-tau217 (threonine tau 217) increase in AD and provide a better diagnostic performance in differentiating AD from non-AD dementias than CSF P-tau181 [133]. The levels of CSF P-tau217 had a stronger correlation with CSF and positron emission tomography (PET) measures of cortical amyloid deposition than did CSF P-tau181 [131].

Levels of the blood-based biomarker, plasma P-tau181, increase early in AD, differentiating AD from cognitively normal controls and other dementias [131]. Plasma P-tau181 levels also increase with AD disease progression over time and predict progression to AD dementia in individuals with mild cognitive impairment (MCI). Plasma P-tau217 has received attention as a blood-based biomarker for AD [134]. 

In contrast to AD, MS affects the entire CNS, without predictable insight into which region of the CNS will be affected in each MS patient. Thus, a form of tau phosphorylated at a specific site might not be found.

Novel assays, using immunoprecipitation coupled with mass spectrometry or SIMOA, allow the measurement of plasma Aβ with high precision and demonstrate the ability of plasma Aβ40/Aβ42 levels to accurately predict amyloid-positive PET scans in cognitively normal or impaired individuals. The SIMOA platform has also been used successfully for plasma P-tau181 measurements, demonstrating the ability of plasma P-tau181 to accurately predict increased brain amyloid and tau on PET scans [30]. For subclinical neurodegeneration in MS, ideally, biomarkers would also need to correlate with changes at the CNS level, indicating neurodegenerative events. 

## 5. N-Acetylaspartate

N-Acetylaspartate (NAA) is an abundant amino acid (AA) synthesized in neurons [13,135]. Several functions for this AA have been postulated, including working as an osmolyte important for the removal of water from neurons. Acetate is also important for myelin synthesis and is a mitochondrial energy source. Acetate is a precursor for N-acetyl aspartyl glutamate and a ligand for glutamate receptors [13]. Reduced acetate levels have been found in MS lesions. Of interest, glatiramer acetate, widely used to treat MS, increases NAA levels in MS lesions [136]. CSF NAA levels decrease during axonal degeneration and disease progression [13,135,136,137]. In addition, Narayanan et al. [137] showed that NAA increases in interferon (IFN) treated vs. untreated groups, suggesting that IFN reverses, in part, axonal injury during MS. It is imperative to investigate whether drugs aimed at neuroprotection increase NAA. Of interest, NAA is a marker of disability in secondary progressive MS as shown in a proton MR spectroscopic imaging study [138]. 

## 6. 14-3-3 Family Proteins

14-3-3 family proteins are highly concentrated in the brain and are expressed in the cytoplasmic and nuclear regions of neurons and glia [13]. These proteins regulate a variety of intracellular processes by interacting with hundreds of target proteins. 14-3-3 proteins are also molecular chaperones with anti-apoptotic effects. The presence of 14-3-3 protein in the CSF establishes Creutzfeldt-Jakob disease, but 14-3-3 protein is also detected in the CSF during other prion-unrelated conditions associated with CNS tissue damage. In MS, CSF, 14-3-3 protein levels correlate with a higher relapse rate and more severe disability, predicting permanent neurological disability after an acute episode [13,139,140,141,142]. No study to date has studied how neuroprotective drugs regulate 14-3-3 protein levels. 

## 7. Additional Biomarkers of Neurodegeneration

Contactin-1 and contactin-2 in cerebrospinal fluid are potential biomarkers for axonal dysfunction in MS [143,144]. Contactin 1, a cell adhesion molecule, is a glycosylphosphatidylinositol-anchored neuronal membrane protein [145], and Contactin-2 is a cell adhesion molecule critical for neuronal patterning and ion channel clustering [146]. Recent evidence has shown the importance of MiR-142-3p for synaptopathy-driven disease progression in MS [147]. Previous studies have also shown glial activation markers in CSF and serum from patients with PP-MS. The authors suggested that serum glial fibrillary acidic protein (GFAP) is a potential marker for disease severity progression [148,149]. Further, monitoring reactive chemical species, oxidative enzymes, antioxidative enzymes, and degradation products might identify the redox status of MS patients [150], and thiol homeostasis may be useful to monitor disease activity [151]. The role of low levels of vitamin D in MS progression is an area of intense research, thus, serum levels of 25-hydroxyvitamin D should also be monitored during MS disease [152].

## 8. Osteopontin

Osteopontin (OPN) is an extracellular matrix glycol-phosphoprotein with a role in autoimmune-mediated demyelinating diseases during multiple sclerosis. OPN regulates inflammatory and regenerative processes during various diseases of the nervous system. OPN concentrations are increased in CSF during active multiple sclerosis [153,154]. 

## 9. Future Directions

The perspective of serum-based biomarkers described in this review must be combined with additional diagnostics. Work by LoPresti [33,155] indicated that an early defect at the level of HDAC6 is present in an animal model of MS. Serum-biomarkers should be added to in vivo HDAC6 changes. HDAC6 human brain mapping with [^18^F] Bavarostat as a radiotracer has been proposed [156] and could be added to serum-based biomarkers to define MS types based on neurodegeneration, subgrouping MS into those patients with early or late neurodegeneration.

This review offers a new approach to elucidate a set of neurodegeneration biomarkers at two distinct time points. The aim is to identify distinct biomarker signatures in early and late subclinical cognitive decline. Newly identified biomarkers could identify the transition between early and late subclinical cognitive decline. For example, recent evidence pointed to GFAP as an important biomarker for progressive MS [148,149], as increased GFAP levels could help to delineate the transition into the late phase of subclinical cognitive decline, preceding progressive MS. Another approach could be the analysis of non-coding RNAs (ncRNAs). Joilin et al. [157] identified a potential ncRNA biomarker signature for ALS. In MS, a potential area of interest could include ncRNAs that distinguish disease activity from cytoskeletal disruption. 

The biggest challenges in MS are two-fold; first, immune-inflammatory alterations during the disease are specific to each patient. Second, any area of the CNS can be a target of the disease. Therefore, by extending the concept of personalized medicine, we propose that the biomarker signature must be personalized for each patient. For example, standard assays should establish the baseline signature of selective biomarkers for each patient, and the evolution of such signature over time would be monitored. The signature shift could indicate progression along with subclinical cognitive decline, bringing that specific patient closer to developing progressive MS.

## 10. Summary

Focusing on neurons in MS patients, synaptic dysfunction and neurodegeneration should be measured so therapeutics can be planned to target each of these problems. Since tau protein is present at the synapses, a specific change in tau posttranslational modification might reveal a synaptic dysfunction, whereas large amounts of tau could indicate tau release due to neurodegeneration. Initial studies in mouse models of MS will highlight the relative importance of a set of serum-based biomarkers that characterizes synaptic dysfunction and neurodegeneration at early, mid, and late stages. In human samples, we expect distinct biomarker profiles to be identified in apparently similar types of MS. In particular, analysis of the blood of MS patients will show that each patient has a unique pattern, irrespective of their apparent clinical type, providing an educated rationale for intervention with selective therapeutics to modify the synaptic or neurodegeneration component. As the ability to distinguish synaptic dysfunction and neurodegeneration is established in each patient, a new understanding of the disease and therapeutic horizons can be envisioned.

## 11. Outstanding Questions

a. Would a new classification of MS types have to include silent progression? 

b. Within clinically similar MS types, could we identify separate entities based on biomarkers of synaptic dysfunction and neurodegeneration? 

c. Can a biomarker-based diagnostic test determine 1. The rate of subclinical memory decline, and 2. The time before the transition from subclinical to clinically apparent neurodegeneration? 

## Figures and Tables

**Table 1:** Neurofilament light is detected in various types of MS patient samples. Neurofilament light is detected in various types of human samples. The detection methods use a variety of assays as indicated, including ELISA, ECL, and SIMOA [65,68,82,83,84,85,86,87,88,89,90,91,92,93,94,95,96,97,98,99,100,101,102,103,104,105,106,107,108,109,110,111,112,113].

Sample	Detection	References
Serum	ELISA	[65]
	ECL	[82,83]
	SIMOA	[84,85,86]
Blood	SIMOA	[68]
CSF	ELISA	[87,88,89,90,91,92,93,94,95,96,97,98,99,100,101,102,103]
Plasma	SIMOA	[104]
Serum + CSF	ELISA	[105,106]
	ECL	[107]
	SIMOA	[108,109,110,111,112,113]

## Abbreviations

AA:	Amino acid
AD:	Alzheimer’s disease
ALS:	Amyotrophic lateral sclerosis
APP:	Amyloid precursor protein
CNS:	Central nervous system
CSF:	Cerebrospinal fluid
EAE:	Experimental autoimmune encephalomyelitis
ECL:	Electro chemi luminescence
ELISA:	Enzyme-linked immunosorbent assay
GFAP:	Glial fibrillary acidic protein
IFN:	Interferon
LBD:	Lewy body dementia
MCI:	Mild cognitive impairment
MRI:	Magnetic resonance imaging
MS:	Multiple sclerosis
NAA:	N-Acetylaspartate
NFs:	Neurofilaments
NFL:	Neurofilament light
PET:	Positron emission tomography
PP-MS:	Primary progressive multiple sclerosis
RR-MS:	Relapsing-remitting multiple sclerosis
SIMOA:	Single-molecule array
sNF:	Serum neurofilament
SP-MS:	Secondary progressive multiple sclerosis
